# Automated Cough Analysis with Convolutional Recurrent Neural Network

**DOI:** 10.3390/bioengineering11111105

**Published:** 2024-11-01

**Authors:** Yiping Wang, Mustafaa Wahab, Tianqi Hong, Kyle Molinari, Gail M. Gauvreau, Ruth P. Cusack, Zhen Gao, Imran Satia, Qiyin Fang

**Affiliations:** 1 Department of Engineering Physics, McMaster University, Hamilton, ON L8S 4K1, Canada; wangy156@mcmaster.ca (Y.W.);; 2 Department of Medicine, McMaster University, Hamilton, ON L8S 4K1, Canada; wahabm1@mcmaster.ca (M.W.); gauvreau@mcmaster.ca (G.M.G.); cusackruth@hotmail.com (R.P.C.); satiai@mcmaster.ca (I.S.); 3 School of Biomedical Engineering, McMaster University, Hamilton, ON L8S 4K1, Canada; hongt6@mcmaster.ca; 4 W Booth School of Engineering Practice & Technology, McMaster University, Hamilton, ON L8S 4K1, Canada; gaozhen@mcmaster.ca

**Keywords:** chronic cough, machine learning, neural network, cough challenge

## Abstract

Chronic cough is associated with several respiratory diseases and is a significant burden on physical, social, and psychological health. Non-invasive, real-time, continuous, and quantitative monitoring tools are highly desired to assess cough severity, the effectiveness of treatment, and monitor disease progression in clinical practice and research. There are currently limited tools to quantitatively measure spontaneous coughs in daily living settings in clinical trials and in clinical practice. In this study, we developed a machine learning model for the detection and classification of cough sounds. Mel spectrograms are utilized as a key feature representation to capture the temporal and spectral characteristics of coughs. We applied this approach to automate cough analysis using 300 h of audio recordings from cough challenge clinical studies conducted in a clinical lab setting. A number of machine learning algorithms were studied and compared, including decision tree, support vector machine, k-nearest neighbors, logistic regression, random forest, and neural network. We identified that for this dataset, the CRNN approach is the most effective method, reaching 98% accuracy in identifying individual coughs from the audio data. These findings provide insights into the strengths and limitations of various algorithms, highlighting the potential of CRNNs in analyzing complex cough patterns. This research demonstrates the potential of neural network models in fully automated cough monitoring. The approach requires validation in detecting spontaneous coughs in patients with refractory chronic cough in a real-life setting.

## 1. Introduction

Cough is one of the most common reasons for seeking medical attention [1]. Many patients experience acute coughing lasting up to three weeks after a viral respiratory tract infection. Approximately 10% of the population suffers from chronic cough (>8 weeks), with detrimental effects on quality of life [2]. Recent data from the Canadian Longitudinal Study of Aging (CLSA) suggest that the prevalence of chronic cough is 16% in adults over the age of 45 and is twice as common in females as in males [3]. The typical symptom is described as a non-productive cough often occurring in distressing bouts, which the patient cannot control, and is usually preceded by an irresistible urge to cough associated with an irritating sensation in the throat. Cough can be precipitated by innocuous triggers such as changes in temperature, strong smells from perfumes or aerosols, dust, and passive smoking [4]. Chronic cough has major psychological, social, and physical consequences [5]. Individuals with chronic cough are embarrassed socially and in the workplace, e.g., by frequent prolonged coughing bouts or even stress incontinence. The treatment options for chronic cough are limited. Over USD 41 billion was spent worldwide in 2023 on over-the-counter cough and cold medications [4], most of which contain dextromethorphan, which has very limited if any clinical effectiveness [6].

Chronic cough can occur in common respiratory conditions such as asthma [7] and chronic obstructive pulmonary disease (COPD) [8]. Other causes include eosinophilic bronchitis, interstitial lung diseases, bronchiectasis or the use of angiotensin-converting enzyme (ACE) inhibitors. Extra-pulmonary diseases such as gastroesophageal reflux disease (GERD) and post-nasal drip secondary to rhinosinusitis can also be identified as potential triggers. After numerous investigations, some people exhibit no evidence of any of the above causes, and their condition is hence classified as “unexplained chronic cough”. In circumstances where there is an objectively identifiable cause, such as asthma or COPD, coughing is often disproportionate to or independent of levels of inflammation or lung function abnormalities [9]. These cases are often referred to as “refractory chronic cough”, i.e., not controlled by current treatment strategies, making them among the most common reasons for referral to a specialist in secondary care [10].

Management of cough relies on accurate diagnosis and continued monitoring of treatment responses from individual patients. It is essential to assess cough severity, treatment effectiveness, monitor disease progression in clinical practice, and evaluate novel anti-tussive drugs in clinical trials.

Patient-reported outcome measures, such as the Leicester Cough Questionnaire, Cough Quality of Life Questionnaire, and Cough Severity Diary, are used in clinical trials to assess health-related quality of life (QoL) [11]. An alternative strategy to assess the severity of cough is to ask patients to rate the severity of their cough on a numerical scale: 0–10, mBorg Scale, or a 100 mm Visual Analogue Scale. However, these techniques are highly subjective hence there is a lack of evidence supporting their utility. They also present the difficulty of comparing subjective scores among individuals [12].

Research on cough “detection” has been an active area, especially since COVID [13]. Researchers are addressing measurement shortcomings in this field by improving signal processing algorithms, enhancing wearable technology, and integrating multi-modal data, while also utilizing telemedicine for continuous patient monitoring [14].

Most studies have focused on detecting the initial event of a cough episode, typically the loudest cough in a series of declining individual coughs. In clinical practice and clinical trials, however, patients have been found to undergo longer episodes of coughing, with the later coughs demonstrating the lowest audio signal amplitude, but requiring the greatest respiratory muscle activation. These episodes correlate the most with cough-related quality of life and severity [15]. These low-amplitude coughs can easily be missed by a smartphone without a separate microphone mounted on it [16].

There are currently no approved tools in clinical practice to objectively assess cough frequency. Nonetheless, objective cough monitoring is used in clinical trials (e.g., the VitaloJAK and the Leicester Cough Monitor). The VitaloJAK monitor is currently the gold standard in cough monitoring and has been used in phase II and III clinical trials for refractory chronic cough [17]. However, it is not used in clinical practice because it is expensive, labor-intensive (requires manual human tagging), and currently is used to measure cough for a 24–48 h period [18]. Its utility for real-time analysis is limited counting is semi-automated and recordings are generally for a 24–48 h period in a research setting, but individuals with chronic cough may experience significant day-to-day variability in cough frequency [19]. Recently, advancements in chronic cough technology have emerged, with the introduction of the Hyfe and Siva cough monitors being used in clinical trials [20]. These digital tools use artificial intelligence (AI) and smartphones to detect and analyze coughs in real time. They provide continuous monitoring outside clinical settings, helping to manage and understand cough patterns in individuals with chronic cough or respiratory conditions. Nonetheless, current methods, including acoustic devices, wearable sensors, and spirometry, face challenges such as noise interference, discomfort, and accuracy issues [21].

Several machine learning (ML) techniques have been applied to cough detection, each with its own strengths and limitations. Support vector machines (SVMs) have been used for classifying cough sounds based on features extracted from audio signals [22]. Random Forests (RFs) is another approach that has shown promise by using ensemble learning to improve classification accuracy [23]. Additionally, traditional ML methods such as Hidden Markov Models (HMMs) have been employed to model the temporal patterns in cough recordings [24]. These techniques offer various ways to handle and analyze cough data, but have not yet been systematically compared in a controlled setting.

More recently, convolutional recurrent neural networks (CRNNs) have emerged as a powerful technique for time-series data analysis, such as recorded audio data [25]. CRNNs combine the strengths of convolutional neural networks (CNNs), which excel at processing spatial data, with recurrent neural networks (RNNs), which are adept at handling sequential information. This integration makes CRNNs particularly well suited for analyzing complex patterns in sequential data like audio. However, a comprehensive study comparing these machine learning techniques for chronic cough detection remains to be conducted.

Therefore, there is a need for non-intrusive and fully automated cough monitors that can measure cough frequency for longer than 24 h and be validated for use in clinical practice and clinical trials. To achieve this goal, new audio data acquisition hardware systems and algorithms are needed. In this paper, we report the development of a CRNN-based deep learning model, which is optimized for detecting and clarifying cough events from audio recordings from a controlled clinical study. The performance of the resulting model is then validated against this dataset and compared to the performance of a few machine learning algorithms.

## 2. Methods

### 2.1. General Methodology

**Clinical Study and Data Collection**: This study was conducted on a cohort of 71 subjects, each undergoing cough challenges to mannitol or capsaicin either twice or three times on separate days. The repeated visits were aimed at capturing comprehensive data points to ensure robustness in the subsequent analysis. These visits allowed us to monitor changes over time, providing a longitudinal dataset critical for our research.
**Preprocessing Pipeline**: The raw audio files collected during the study visits underwent a preprocessing pipeline to convert them into a format suitable for machine learning analysis. Specifically, the audio data were transformed into Mel spectrograms, which are widely used in audio processing due to their ability to represent the power spectrum of a signal. This step is crucial, as it standardizes the data and extracts relevant features necessary for training machine learning models.
**Machine Learning and Neural Network Models**: Following preprocessing, the Mel spectrogram dataset was used to train various machine learning and neural network models. We employed both traditional machine learning algorithms and more advanced neural network architectures to ensure a comprehensive analysis. The models were selected based on their relevance to the problem at hand and their ability to handle the complexities of the dataset. Detailed descriptions of the model architectures, training parameters, and optimization techniques are provided in the subsequent sections.
**Model Testing and Evaluation**: After training, each model was tested on the preprocessed dataset. The testing phase involved evaluating the models’ performance using various metrics to ensure accuracy, generalizability, and robustness. A comparative analysis of the models’ results is presented to highlight the strengths and weaknesses of each approach, providing insights into the most effective methods for this particular application.

### 2.2. Study Protocol Design, Ethics, Data Collection and Description

In the study, anonymized audio data collected from three clinical studies using similar cough challenge protocols were used to train and test the cough classification models. These studies followed protocols consistent with good clinical practice (GCP) according to International Conference for Harmonisation (ICH) guidelines. The protocols were reviewed and approved by the Hamilton Integrated Research Ethics Board (HiREB). The studies are (participant information summarized in Table 1).

1. The effect of sex hormone levels during the menstrual cycle on capsaicin-evoked cough responses in mild allergic asthmatics (CASH)—HiREB Project #7343.
2. Mannitol-Induced Cough Challenge in Healthy Controls and Subjects with Mild Allergic Asthma (MICC)—HiREB Project #4657.
3. The effects of salbutamol on mannitol-induced cough responses in healthy controls (COMA)—HiREB Project #11537.

The first study was a prospective observational study of 2 capsaicin cough challenges repeated during different phases of the menstrual cycle in female participants with allergic asthma. The 2nd and 3rd studies enrolled male and female participants in randomized controlled studies where mannitol cough challenges were conducted on 3 different occasions (baseline, pre-placebo, and pre-salbutamol). All participants went through the informed consent process and provided their consent for these studies. The data used in this study are anonymized without any identification.

All three studies were independently conducted in a clinical laboratory setting at the McMaster University Medical Center by medically qualified persons. Participants inhaled mannitol or capsaicin as a tussive agent in a dose–response manner as described previously [26] (i.e., the coughs are experimentally evoked by the inhalational agent). Participants wore the VitaloJAK cough monitor for the duration of the cough challenge, which lasted approximately 1 h. After each inhalation, the number of coughs was counted by the investigator performing the cough challenge and later verified by listening to the audio file. The clinical lab was in a low-personnel-traffic area, but may have had more than one participant under observation at the same time.

### 2.3. Data Acquisition

**Figure 1:**
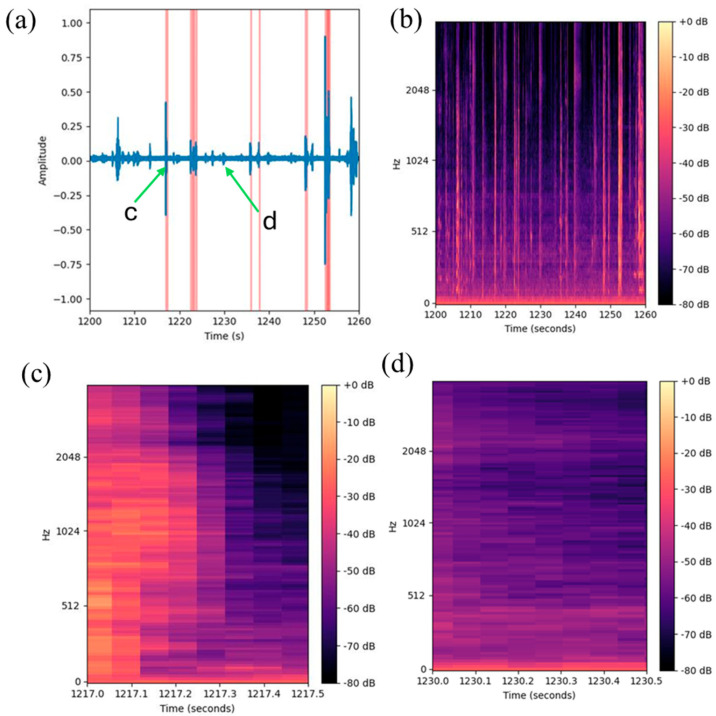
Examples of cough data in time (**a**)- and frequency (**b**–**d**)-domain formats: (**a**) 2 min amplitude graph, with coughing events marked by red lines; (**b**) Mel spectrogram of the same audio segment; (**c**) 0.5-s audio segment containing a cough occurring between 1217.0 and 1217.3 s; (**d**) 0.5-s audio segment that does not contain a cough. The color bar shows volum of the recording by decibel from yellow (0 db) to black (80 db).

To establish the training pipeline, we collected a cough challenge dataset. The one-hour audio recording was retrieved and saved at the end of each study session. The recordings were a time-series sequential dataset that could be visualized using waveform of the recorded audio data (Figure 1a) [27]. The goal of the classification was to predict whether a given segment of a recording contained a cough.

The audio files were in .wav format with a sampling rate of 22,050 Hz, and each file varied in length, ranging from 30 to 50 min. For each .wav file, there was a corresponding .txt file that annotated special events (such as coughing, laughing, or throat clearing) with their start and end times.

### 2.4. Data Annotation

Coughing is a physically observable phenomenon that can be recognized by an experienced listener. Coughs are typically identifiable by sudden and short-amplitude spikes, as illustrated in Figure 1. The highlighted areas mark the coughing event, and the wider areas indicate multiple consecutive coughs. To establish ground truth and for performance evaluation in the subsequent classification, manual annotation of the recorded audio data was performed by a number of trained listeners using the digital audio processing software Audacity. Individual cough and cough-related sound events (e.g., speech, laugh, throat clearing, etc.) were also tagged with a time stamp.

The recorded audio contained long periods of silence (Figure 1a). A threshold was applied to the recorded audio data, and the silent segments were removed. This step reduced the total length of each recording from 60 min to 10–15 min, which significantly alleviated the effort of manual annotation. In the classification model training and testing, the original dataset was used without removing the silent segments.

### 2.5. Data Preprocessing

The goal of this project was to identify individual coughs, which are usually shorter than 1 s. The audio data stream was analyzed using a 0.5-s sliding window, which is commonly used in sequential analysis studies [28].

For machine learning applications, raw audio waveform data are not preferred for analysis. Mel spectrograms are a time-frequency representation of audio signals that are particularly useful for analyzing and interpreting sound data. They transform a time-domain audio signal into a two-dimensional representation where one axis represents time and the other represents frequency [29]. The Mel spectrogram is constructed by applying a short-time Fourier transform (STFT) to the audio signal to obtain the spectrogram, followed by a Mel filter bank to map the frequency axis to the Mel scale, which approximates the human ear’s perception of pitch more accurately than the linear frequency scale [30].

This Mel scale compresses higher frequencies and expands lower frequencies, aligning with human auditory sensitivity. In the context of cough detection and other audio analysis tasks, the Mel spectrogram is a valuable feature due to its ability to capture both temporal and spectral characteristics of the sound [31]. It provides both the volume and frequency of the sound, which can be used for classification. Cough sounds often exhibit complex patterns and subtle variations that can be challenging to detect with raw audio data alone. By converting these sounds into Mel spectrograms, it becomes easier to apply machine learning algorithms, such as convolutional neural networks (CNNs) and convolutional recurrent neural networks (CRNNs), which excel in processing image-like data and sequential patterns. The Mel spectrogram provides a more structured and informative representation of the audio signal, enhancing the accuracy of cough detection systems by enabling these algorithms to learn and identify distinctive features and patterns associated with different types of coughs.

The audio data were converted into Mel spectra as 2D arrays, similar to an image, as exemplified in Figure 1. The horizontal axis corresponds to the length of the audio segment, while the vertical axis represents the different frequency channels. After processing, each 0.5-s segment was transformed into an image of size 128 × 22.

The original dataset was imbalanced: the proportion of positive samples (segments containing cough) was lower than 5%. An imbalanced dataset can lead to a model that struggles to detect coughs effectively [32]. This happens because the model tends to learn to ignore coughs altogether, favoring the more frequent non-cough segments, which skews the results and reduces the model’s accuracy in identifying the coughs.

To refine the data, we initially filtered based on amplitude, operating under the assumption that cough sounds are often higher in amplitude compared to other sounds such as speech, throat clearing, or background noise. However, this approach may not always be reliable, especially during latter coughs in a coughing bout, where the amplitude tends to decrease, despite being a part of the same coughing event. Therefore, relying solely on amplitude as a criterion may result in missing significant cough instances, particularly towards the end of a coughing episode. By calculating the average amplitude of the entire raw audio and comparing it against each 0.5-s segment, segments with average amplitude lower than the global average were removed unless they contained cough (identified manually in the ground truth), in which case they were retained. After this step, the segments containing cough made up 7.4% of all segments, which was suitable for the machine learning training step. For the development of classification algorithms where the ground truth is known, this approach is effective in removing negative results in an imbalanced training dataset.

### 2.6. Feature Extraction

In this study, several features were extracted from the audio data to enhance the accuracy of cough detection. These features included Mel spectrograms, which capture the spectral and temporal characteristics of cough sounds, and Mel frequency cepstral coefficients (MFCCs), which are commonly used in audio processing to represent the short-term power spectrum of sound. Additionally, zero-crossing rate (ZCR) was used to measure the rate at which the signal changes sign, and spectral roll-off was included to capture the frequency below which a certain percentage of the total spectral energy was contained.

### 2.7. Neural Network Model

Considering the time-series nature of our data, we adopted a neural network approach, focusing on models that are well suited for handling sequential data. Note that the dataset used to train the CRNN did not undergo the threshold preprocessing steps applied to the traditional ML models.

**Figure 2:**
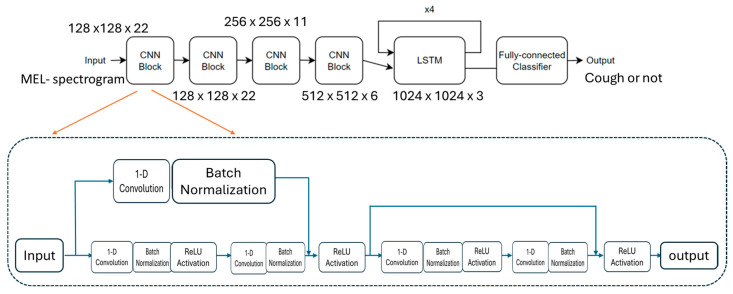
The structure of the CRNN model.

We introduced a residual connection between the input and the first convolution layer within each convolution block. Specifically, after passing through a simple layer, the input signal is added to the output of the first convolution layer. This residual design helped deep layers retain important features, enhancing our model’s ability to learn complex patterns. By transforming the input before adding it back, the network can adapt more flexibly to different types of data, improving both performance and stability. Additionally, we implemented four feed-forward convolution blocks along with a four-layer long short-term memory (LSTM) model. This design choice offers a good balance between computational efficiency and model complexity. At the top, there are four convolutional neural network (CNN) blocks that function as encoders, compressing each sample into a more compact representation. The first CNN block contained 128 filters, and each subsequent block doubled the number of filters, culminating in the fourth CNN block, which produced 1024 channels (128 × 8). This encoded representation was then passed to a four-layer LSTM block, where the input size was 1024 and the hidden layer size was 512. Finally, the output from the LSTM block was sent into a fully connected layer, which produces the final prediction. The architecture of our CRNN model is shown in Figure 2.

Our dataset remained imbalanced, with a 92% negative ratio. To address this, we implemented a weighted loss function in our neural network. This approach assigns a larger weight to the positive class during backpropagation, causing the model to incur a significantly higher loss when it fails to recognize a cough compared to when it mistakenly predicts a non-cough segment as a cough. Let the number of positive samples be *n_true_* and the number of negative samples be *n_false_*, then the weight for positive-class *w_true_* is calculated by:

(1) $$w_{true}=\frac{n_{true}}{n_{false}}$$

The weighted loss function becomes:

(2) $$L_{weighted}=-\left(w_{true}*y*\log\left(p\right)\right)+\left(1-y\right)*log(1-p)$$

where *L_weighted_* is the loss, *y* is the label, and *p* is the model’s prediction probability of the class being true.

### 2.8. Performance Metrics and Comparison

We experimented with several conventional machine learning (ML) approaches, including decision tree, random forest, linear regression, and support vector machine (SVM). Prior to training the ML models, the dataset was subjected to a standard preprocessing pipeline. This pipeline included flattening the 2D spectrogram segments into 1D arrays, transforming them from (128, 22) to (2816), which was too high to process. Principal component analysis (PCA) was then applied to reduce the dimensionality of the data from 2816 to 326 components while retaining 95% of the variance. Following this, the dataset was normalized to achieve zero mean and unit variance. After this preprocessing, each data segment was represented as a 326-component 1D array. It is important to note that this preprocessing approach was specifically designed for the conventional ML models used in this study.

In our experiments, we utilized several machine learning models with default hyperparameters from scikit-learn. The logistic regression model employed an L2 penalty, C = 1.0, max_iter = 1000, and the lbfgs solver. For the decision tree classifier, we used the criterion = “entropy”, with no depth limit (max_depth = none), and the minimum sample split was set to 2. The Random Forest Classifier was configured with n_estimators = 100, criterion = “entropy”, and random_state = 42. Lastly, the support vector classifier (SVC) utilized an RBF kernel with C = 1.0, gamma = “scale”, and degree = 3. All other hyperparameters were left at their default settings [33].

To evaluate the performance of our models, we used the following performance metrics:

(3) $$Accuracy=\frac{TP+TN}{TP+TN+FP+FN}$$

(4) $$Specificity=\frac{TN}{TN+FP}$$

(5) $$Recall\;\left(Sensitivity\right)=\frac{TP}{TP+FN}$$

where:

True Positive (*TP*): Segment contains cough, and the model predicts a cough.
True Negative (*TN*): Segment does not contain cough, and the model predicts there is no cough.
False Positive (*FP*): Segment does not contain cough, and the model predicts there is a cough.
False Negative (*FN*): Segment contains cough, and the model predicts there is no cough.

## 3. Results

We trained and tested the aforementioned conventional ML models and the CRNN model on the CCH dataset. Some algorithms perform better than others, because linear models like linear regression works well for simple, linear relationships, while decision trees and random forests handle complex, non-linear interactions better. Random forests and SVMs generally offer improved generalization by reducing overfitting and handling high-dimensional data effectively, albeit at the cost of increased computational complexity. Ultimately, the best-performing algorithm is context-dependent, considering factors like data structure, noise, and the need for interpretability. Our CRNN model outperformed all traditional machine learning models, achieving the best results with superior sensitivity. Recall that our training dataset was imbalanced with a positive rate of 7.4%. Therefore, the baseline accuracy was 92.6%, which is the proportion of negative samples. This means that if the model blindly predicts “false” for all samples, it will still achieve an accuracy of 92.6%. All models are expected to perform better than baseline accuracy. Table 2 shows the performance of all models. All conventional machine learning models tested here showed poor sensitivity. Although they may have high accuracy, their low sensitivity value is not favored in medical diagnosis. The CRNN model had much better sensitivity compared to the rest, while maintaining satisfactory accuracy. Note that the accuracy, sensitivity, and specificity were calculated by testing the models on the untrained 10% of the dataset.

Our training dataset consisted of audio recordings from three different environmental settings. Cross-validation is a widely used technique for assessing how well a machine learning model adapts to different scenarios, represented here by these three smaller datasets. By training and testing the model on each dataset, cross-validation helps understand how well a model trained on one scenario can generalize to another, which shows the model’s real-world viability where data might vary in unforeseen ways.

**Figure 3:**
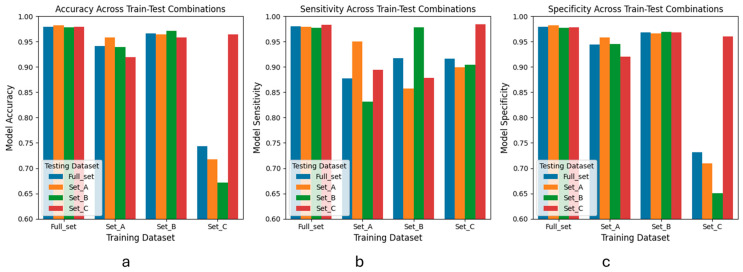
Cross-validation results of models trained and tested on different datasets. The metrics used were accuracy, sensitivity, and specificity. Blue represents full data set, orange represents set A, green represents set B and red represents. (**a**) Accuracy across train-test combinations. (**b**) Sensitivity across train-test combinations. (**c**) Specificity across train-test combinations.

We cross-validated the CRNN model (Figure 3) and found that the accuracy of training on subset C was significantly lower than when training on the other subsets, although the sensitivity metric remained stable. This suggested that subset C may have had significant differences from the other datasets and a model trained on subset C tended to be more “conservative”, with a higher likelihood of classifying non-coughs as coughs. This evidence indicates that the environment and audio recording setting can heavily impact the model’s performance, particularly if the model has not been exposed to data from other settings.

**Figure 4:**
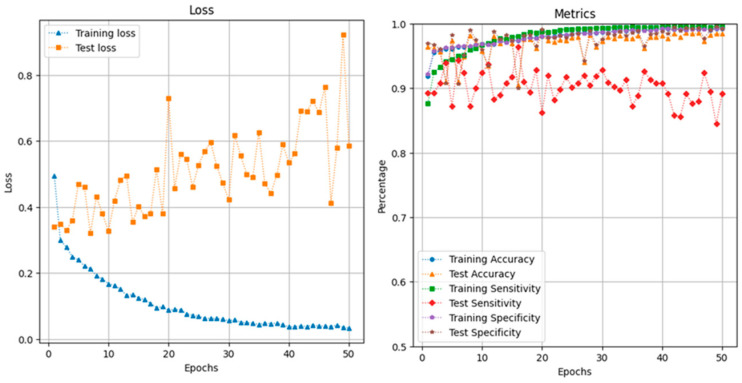
Training and testing performance metrics of the CRNN model across 50 epochs. Left figure bule line shows the training loss and orange line shows the test loss. Right figure blue line shows the training accuracy, orange shows test accuracy, green line shows the training sensitivity, red line shows the test sensitivity, purple line shows the training specificity and brown line shows the test specificity.

In medical testing, there is a classic trade-off between accuracy and sensitivity. A model that predicts more positives to increase sensitivity might also increase the number of false positives, potentially lowering accuracy if negatives are misclassified more frequently. During the training of the CRNN model, we observed this trade-off pattern (Figure 4). The accuracy and sensitivity of the CRNN on the test dataset oscillated and showed an inverse relationship: an increase in accuracy almost always resulted in a decrease in sensitivity and vice versa. Additionally, our model suffered from overfitting, where the test loss increased as the training loss decreased. This indicated that the model performed well on the training data, but failed to generalize to unseen data, capturing noise and patterns specific to the training set, rather than the underlying distribution. Overfitting may also contribute to the accuracy–sensitivity trade-off issue.

One workaround for the trade-off issue is to save the CRNN model parameters at three different time stamps during a single training run: one when the model achieves the best accuracy, one when it achieves the best sensitivity, and one with the best mixed performance. The metric for mixed performance is calculated by simply adding accuracy and sensitivity, providing a better overall performance evaluation.

- When the CRNN achieved its best accuracy of 0.98, its sensitivity was 0.89.
- When the CRNN achieved its best sensitivity of 0.96, its accuracy was 0.90.

**Figure 5:**
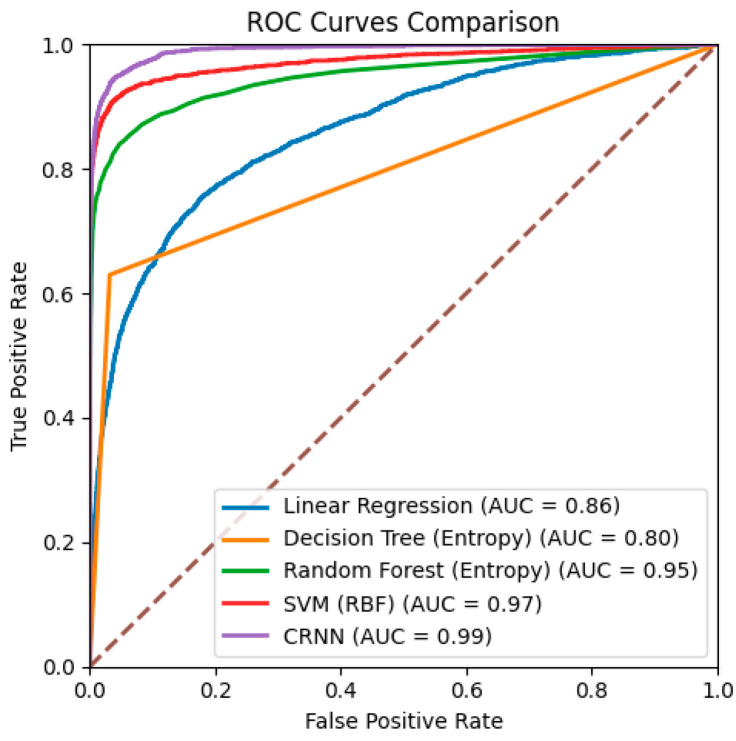
ROC curves of all four machine learning modes and the CRNN model. All models were trained and tested on the filtered CCH dataset. The ROC curve of CRNN is from the best mixed-performance model.

The best mixed-performance CRNN has an accuracy of 0.97 and a sensitivity of 0.92. We also plotted receiver operating characteristic (ROC) curves for all models (Figure 5), which demonstrated that our CRNN model achieved a higher true-positive rate while maintaining a lower false-positive rate across various thresholds. Additionally, our CRNN model has the highest area under the curve (AUC) of 0.984 among all machine learning models.

Despite the trade-off and overfitting issue, our CRNN model still achieved accuracy of 97.2%, sensitivity of 92.9%, and specificity of 97.6% during its training run. These results are promising for the deployment of real-time cough detection systems.

## 4. Discussion and Conclusions

This study presents a machine learning approach to addressing the challenges of chronic cough monitoring by developing an automated system using a convolutional recurrent neural network (CRNN). Chronic cough poses a significant burden to patients and healthcare professionals, with limited effective tools for continuous, objective monitoring. Traditional methods, including subjective patient-reported outcomes and limited-use devices like the VitaloJAK monitor, currently do not provide continuous analysis or work beyond 24 h. This CRNN model outperformed traditional machine learning approaches and demonstrated sufficient accuracy in detecting cough events from audio recordings. Our methodology offers a scalable automated solution for long-term cough monitoring in clinical and real-world settings, showing potential for product deployment and commercialization.

The results obtained are comparable to those manually labeled using the VitaloJAK monitor, demonstrating a similar level of accuracy. However, these findings are based on cough challenge data collected in a quiet environment during a clinical study setting. To reach the goal of long-term, spontaneous 24/7 recording in daily living settings, the complexity of the audio environment will inevitably be much higher and may differ from this dataset. Therefore, future development using data from daily living settings is necessary, albeit presenting challenges in data curation and privacy concerns, particularly regarding recorded speech, which must be addressed to ensure ethical data collection and usage.

The audio features in the current dataset were extracted using the standard Mel spectrogram technique, which facilitates direct comparisons with other cough detection methods in the literature. This approach is sufficient for the current dataset, as it achieves classification accuracy of 95% or higher. However, in future studies where audio data are collected in more complex, real-world environments, higher-order feature extraction techniques, such as log Mel spectrogram or log–log Mel spectrogram (L2M), could be explored to improve robustness and accuracy [34]

While promising, the model exhibited some overfitting, indicating the need for further improvements. Future work should focus on enhancing model robustness and exploring integration into wearable or mobile technologies. Overall, our findings highlight the potential for AI-driven solutions, offering accurate and continuous monitoring for chronic cough patients.

## Figures and Tables

**Table 1:** Participant summary.

Variable	CASH (N = 31)	COMA (N = 20)	MICC (N = 20)
Disease Group	Asthmatics	Healthy Controls	Asthmatics
Gender (M:F)	11:20	10:10	10:10
Age, Mean (SD)	22.99 (4.48)	25.05 (3.23)	23.20 (9.48)
BMI (kg/m^2^), Mean (SD)	25.08 (4.79)	24.04 (4.83)	23.20 (9.48)

Data presented as means (SD) or geometric means (SD).

**Table 2:** Performance comparison of different machine learning models.

Model Name	Accuracy	Sensitivity	Specificity
Logistic regression (train until converge)	0.93 ± 0.046	0.25 ± 0.013	0.99 ± 0.050
Decision tree (entropy criterion)	0.94 ± 0.047	0.62 ± 0.031	0.96 ± 0.048
Random forest (entropy criterion)	0.96 ± 0.048	0.49 ± 0.025	0.99 ± 0.050
Support vector machine (RBF kernel)	0.98 ± 0.049	0.76 ± 0.038	0.99 ± 0.050
Neural network (CRNN)	0.97 ± 0.049	0.92 ± 0.046	0.97 ± 0.049

## Data Availability

Data is unavailable due to privacy restrictions.

## References

[1] Song W.J., Chang Y.S., Faruqi S., Kim J.Y., Kang M.G., Kim S., Jo E.J., Kim M.H., Plevkova J., Park H.W. (2015). The global epidemiology of chronic cough in adults: A systematic review and meta-analysis. Eur. Respir. J..

[2] Holzinger F., Beck S., Dini L., Stöter C., Heintze C. (2014). The diagnosis and treatment of acute cough in adults. Dtsch. Ärzteblatt Int..

[3] Satia I., Mayhew A.J., Sohel N., Kurmi O., Killian K.J., O’Byrne P.M., Raina P. (2021). Prevalence, incidence and characteristics of chronic cough among adults from the Canadian Longitudinal Study on Aging. ERJ Open Res..

[4] Satia I., Badri H., Al-Sheklly B., Smith J.A., Woodcock A.A. (2016). Towards understanding and managing chronic cough. Clin. Med..

[5] Adams R.J., Appleton S.L., Wilson D.H., Taylor A.W., Ruffin R.E. (2009). Associations of physical and mental health problems with chronic cough in a representative population cohort. Cough.

[6] Bem J.L., Peck R. (1992). Dextromethorphan: An overview of safety issues. Drug Saf..

[7] Smith J.A., Woodcock A. (2016). Chronic cough. N. Engl. J. Med..

[8] Pauwels R.A., Rabe K.F. (2004). Burden and clinical features of chronic obstructive pulmonary disease (COPD). Lancet.

[9] Chung K.F., McGarvey L., Song W.J., Chang A.B., Lai K., Canning B.J., Birring S.S., Smith J.A., Mazzone S.B. (2022). Cough hypersensitivity and chronic cough. Nat. Rev. Dis. Primers.

[10] Shields J.B., Callen E., Loskutova N.Y., Schelfhout J., Hester C.M. (2024). Chronic cough diagnosis, treatment, and referral practices among family physicians in the United States: A survey study. BMC Prim. Care.

[11] Boulet L.P., Coeytaux R.R., McCrory D.C., French C.T., Chang A.B., Birring S.S., Smith J., Diekemper R.L., Rubin B., Irwin R.S. (2015). Tools for assessing outcomes in studies of chronic cough: CHEST guideline and expert panel report. Chest.

[12] Martin Nguyen A., Bacci E.D., Vernon M., Birring S.S., Rosa C.L., Muccino D., Schelfhout J. (2021). Validation of a visual analog scale for assessing cough severity in patients with chronic cough. Ther. Adv. Respir. Dis..

[13] Tena A., Claria F., Solsona F. (2022). Automated detection of COVID-19 cough. Biomed. Signal Process. Control.

[14] Sharma S., Rawal R., Shah D. (2023). Addressing the challenges of AI-based telemedicine: Best practices and lessons learned. J. Educ. Health Promot..

[15] Turner R.D., Birring S.S. (2023). Measuring cough: What really matters?. J. Thorac. Dis..

[16] Eni M., Mordoh V., Zigel Y. (2022). Cough detection using a non-contact microphone: A nocturnal cough study. PLoS ONE.

[17] Spinou A., Birring S. (2014). An update on measurement and monitoring of cough: What are the important study endpoints?. J. Thorac. Dis..

[18] Martin Nguyen A., Bacci E., Dicpinigaitis P., Vernon M. (2020). Quantitative measurement properties and score interpretation of the Cough Severity Diary in patients with chronic cough. Ther. Adv. Respir. Dis..

[19] Georas S.N., Wright R.J., Ivanova A., Israel E., LaVange L.M., Akuthota P., Carr T.F., Denlinger L.C., Fajt M.L., Kumar R. (2022). The Precision Interventions for Severe and/or Exacerbation-Prone (PrecISE) Asthma Network: An overview of network organization, procedures, and interventions. J. Allergy Clin. Immunol..

[20] Kuhn M., Nalbant E., Kohlbrenner D., Alge M., Kuett L., Arvaji A., Sievi N.A., Russi E.W., Clarenbach C.F. (2023). Validation of a small cough detector. ERJ Open Res..

[21] Ijaz A., Nabeel M., Masood U., Mahmood T., Hashmi M.S., Posokhova I., Rizwan A., Imran A. (2022). Towards using cough for respiratory disease diagnosis by leveraging Artificial Intelligence: A survey. Inform. Med. Unlocked.

[22] Chowdhury N.K., Kabir M.A., Rahman M.M., Islam S.M.S. (2022). Machine learning for detecting COVID-19 from cough sounds: An ensemble-based MCDM method. Comput. Biol. Med..

[23] Nguyen T.T., Huang J.Z., Nguyen T.T. (2015). Unbiased Feature Selection in Learning Random Forests for High-Dimensional Data. Sci. World J..

[24] Teyhouee A., Osgood N.D. (2019). Cough detection using hidden markov models. Social, Cultural, and Behavioral Modeling, Proceedings of the 12th International Conference, SBP-BRiMS 2019, Washington, DC, USA, 9–12 July 2019.

[25] Krichen M. (2023). Convolutional neural networks: A Survey. Computers.

[26] Spector S. (2010). Use of mannitol inhalation challenge in assessment of cough. Lung.

[27] Messaoud I.B., Cheikh E.B., Chiboub A., Loulou K., Ouakrim Y., Jebara S.B., Dixon P.C., Mezghani N. Machine Learning Based Approaches for Cough Detection from Acceleration Signal. Proceedings of the 2023 International Conference on Cyberworlds (CW), Sousse, Tunisia, 3–5 October 2023.

[28] Kapetanidis P., Kalioras F., Tsakonas C., Tzamalis P., Kontogiannis G., Karamanidou T., Stavropoulos T.G., Nikoletseas S. (2024). Respiratory Diseases Diagnosis Using Audio Analysis and Artificial Intelligence: A Systematic Review. Sensors.

[29] Darshana S., Rautaray S.S., Pandey M. (2021). AI to Machine Learning: Lifeless Automation and Issues. Machine Learning: Theoretical Foundations and Practical Applications.

[30] Zhang T., Feng G., Liang J., An T. (2021). Acoustic scene classification based on Mel spectrogram decomposition and model merging. Appl. Acoust..

[31] Zhou Q., Shan J., Ding W., Wang C., Yuan S., Sun F., Li H., Fang B. (2021). Cough recognition based on mel-spectrogram and convolutional neural network. Front. Robot. AI.

[32] Kotsiantis S., Kanellopoulos D., Pintelas P. (2006). Handling imbalanced datasets: A review. GESTS Int. Trans. Comput. Sci. Eng..

[33] Antal-Vaida C. (2021). Basic Hyperparameters Tuning Methods for Classification Algorithms. Inform. Econ..

[34] Xia T., Han J., Mascolo C. (2022). Exploring machine learning for audio-based respiratory condition screening: A concise review of databases, methods, and open issues. Exp. Biol. Med..

